# A novel way to understand and communicate the burden of AntiPsycHotic prescribing for adults across specialist Intellectual Disability services in England and Wales: the APHID feasibility study protocol

**DOI:** 10.3389/frhs.2025.1393805

**Published:** 2025-05-09

**Authors:** Emily Stanyard, Helen Neilens, Victoria Allgar, Matthew Bailey, Crispin Musicha, Kiran Purandare, Bhathika Perera, Ashok Roy, Indermeet Sawhney, Lance Watkins, Sujeet Jaydeokar, Sarah Lennard, Sarah Mitchell, Paula McGowan, Richard Laugharne, Samuel J. Tromans, Rohit Shankar

**Affiliations:** ^1^ Peninsula Clinical Trials Unit, University of Plymouth, Plymouth, United Kingdom; ^2^ Medical Statistics Group, University of Plymouth, Plymouth, United Kingdom; ^3^ The Learning Disabilities Directorate, Central & NW London NHS Foundation Trust, London, United Kingdom; ^4^ Department of Psychiatry, University College London, United Kingdom; ^5^ Department of Psychiatry of Intellectual Disability, Coventry and Warwickshire Partnership NHS Trust, Coventry, United Kingdom; ^6^ Learning Disability Team, Hertfordshire Partnership University NHS Foundation Trust, St Albans, United Kingdom; ^7^ Unit for Development in Intellectual and Developmental Disabilities, University of South Wales, Pontypridd, United Kingdom; ^8^ Cornwall Intellectual Disability Equitable Research (CIDER), Peninsula School of Medicine, University of Plymouth, Plymouth, United Kingdom; ^9^ Centre for Autism, Neurodevelopmental Disorders, and Intellectual Disability, Cheshire and Wirral Partnership NHS Foundation Trust, Chester, United Kingdom; ^10^ Chester Medical School, University of Chester, Chester, United Kingdom; ^11^ CIDER, Cornwall Partnership NHS Foundation Trust, Cornwall, United Kingdom; ^12^ Adult Learning Disability Service, Leicestershire Partnership NHS Trust, Leicester, United Kingdom; ^13^ SAPPHIRE Group, Department of Population Health Sciences, University of Leicester, Leicester, United Kingdom

**Keywords:** intellectual disability, learning disability, antipsychotic, psychotropic, overprescribing

## Abstract

**Background:**

The stopping overmedication of people with a learning disability, autism, or both (STOMP) programme was launched in 2016 in response to concerns about the over-prescribing of medication in people with intellectual disability. The programmes focus has been on the withdrawal of antipsychotic treatment for the individual person than the service or dosage optimisation. It could be that cumulative service level antipsychotic treatment converted and presented as chlorpromazine units could allow for comparison of services on how antipsychotic treatment is being utilised and allow for comparing of practices between services in different regions. The aim of this feasibility study is to explore if cumulative service scores of antipsychotic treatment burden could define prescribing patterns across different specialist intellectual disability services in England and Wales, focused on those on ≥2 antipsychotic treatments. There is no evidence to use ≥2 antipsychotic treatments for any individual.

**Methods:**

The study is a feasibility cross-sectional study investigating service antipsychotic treatment cumulative burden at seven annual time points, 2017–2023. De-identified data for adult patients with intellectual disability under the care of specialist intellectual disability services in receipt of ≥2 oral and/or long-acting IM (intramuscular) injectable (depot) antipsychotic treatments are included. Demographic and clinical data will be collated, in addition to information on the prescribed antipsychotic treatments. The data will be evaluated for data completeness and will be inputted into the Statistical Process Control tool. Outcomes will be measured using a combination of methods including descriptive analysis (including mean, standard deviation and percentage values), and a mixed effects regression model, to determine changes in chlorpromazine equivalent dose values over time.

**Results:**

Seven England and Wales National Health Service intellectual disability services are recruiting up to 490 people. There were recognised challenges in identifying the relevant eligible cohort across services and administering a common set of outcome measures.

**Discussion:**

This study is intended to inform decisions to design a wider registry that would involve antipsychotic treatment prescribing data for patients across multiple sites nationwide. Developing a de-identified database using routinely collected data, without the requirement for informed consent, comes with unique benefits and challenges.

**Registry reference:**

NCT06238089 (www.clinicaltrials.gov).

## Introduction

Intellectual Disability (ID) is defined by significant limitations in both intellectual functioning and adaptive behaviour originating prior to adulthood (1). The global prevalence of ID is approximately 1% (2). In the United Kingdom (UK) approximately 17.5% of PwID are prescribed APTs (3). APTs are mainly used to treat mental health conditions such as schizophrenia and other psychoses, agitation, severe anxiety, mania and violent or dangerously impulsive behaviour. The prevalence of psychiatric disorders in those with severe ID is higher than in those with mild or no ID (4) and therefore the use of APTs in some cases is justified. Data from the records of 571 general practices in the UK showed that 71% of PwID treated with APTs did not have a record of severe mental illness (5). APTs can have adverse consequences, both in the short-term (e.g., sedation) and longer-term (e.g., diabetes, obesity). Thus, judicious prescribing is of paramount importance.

In 2016, National Health Service (NHS) England launched the stopping overmedication of people with a learning disability, autism, or both (STOMP) initiative, in response to concerns about overprescribing of psychotropic medications for PwID, particularly, APT, and especially outside the licensed indication for these medications (6).

Prior to the COVID-19 pandemic, national NHS digital data demonstrated a small reduction in APTs prescribed for PwID since the launch of the STOMP initiative (7). However, the COVID-19 pandemic had a profound impact on health outcomes across the world, with PwID experiencing a disproportionate impact (8). PwID often received reduced support relative to pre-pandemic times, with widespread difficulties in accessing medical, mental health, and social support services (9). Preliminary data taken from a limited sample of adults accessing specialist adult ID services across two England-based healthcare trusts suggested that the pandemic led to increased APT prescribing, particularly in areas more significantly impacted by the pandemic (i.e., those associated with a greater level of lockdown restrictions, as well as greater urbanisation) (10). In less specialised settings, a decrease in the prescribing of antipsychotics in PwID was observed (11). However, this did not account for antipsychotics that had been restarted, or doses that had been increased in the time period, or antipsychotics that may have been prescribed by secondary care services (11).

Previous NHS big data analyses of this issue highlight significant concerns but fail to provide a picture of what successful prescribing practices look like, as they tend to be principally focussed on prescribing prevalence rather than dose. APT burdens need to be measured according to medication doses to identify how and what is being prescribed and therefore identify the problems associated. First and second-generation APTs will be evaluated as they cause different side effects. First generation APTs cause significantly more extrapyramidal effects while second generation APTs are considered to have a greater risk of metabolic side effects (12). By recording the specific APTs that have been prescribed, the different prescriber preferences across the country can be examined and understood.

Previous research has tended to report on entire clinical populations of PwID, rather than a specific focus on those in receipt of ≥2 different forms of APTs, who are at greater risk (13). To date there is no clinical rationale or evidence to support two different APTs being prescribed (13). The study aims to determine if it is feasible to quantify multiple (≥2) APT prescribing in PwID as Chlorpromazine equivalent dose values (14), in specialist adult ID services across England and Wales between 2017 and 2023. Such potential data allows the comparison of prescribing patterns (including dosage levels) across specialist adult ID services in the UK over time. The 7-year period of data collection will allow the study of the data prior to the COVID-19 pandemic, during and for a short time after to observe the effects of this on prescribing by clinicians in these services.

Once the prescribing patterns of those receiving ≥2 APTs are further understood, it may allow for the opportunity to progress to obtain further understanding of the patterns across the country for those in receipt of one APT. It may also allow researchers to identify trends and patterns of variation, including measuring the impact of initiatives intended to improve patient care, such as STOMP.

The NHS England Statistical Process Control (SPC) tool (15) is an analytical technique that provides a validated means of plotting data over time. The SPC tool plots variation in data over time in a graphical format, helping better understand the impact of interventions and the most appropriate subsequent actions to take. The SPC tool operates in Microsoft Excel and requires extracted quantitative data to be manually entered, and also enables the user to enter upper and lower limits for expected data variation. This supports easier identification of any outlying data, with the tool also permitting annotation of any significant datapoints in the graphical output. In this study, it will be used to see if it enables researchers to track yearly APT prescribing among PwID receiving multiple forms of APT and monitor variation between services and patient groups. If this proves successful, then the SPC tool could be used to evaluate and measure ID specialist service use of APTs in PwID nationwide.

The study will help determine if mathematic dose conversion of APT and the use of the SPC can provide a readily understandable means to measure APT use across healthcare trusts, as well as within trusts over time. If this method is found to be successful for APT, similar methodology could be used to measure the burden of use of other families of psychotropic medications, such as antidepressants and benzodiazepines. As a result, this could provide a more comprehensive overview of psychotropic medication use among adults under the care of specialist adult ID services.

The study aims to help develop a novel, understandable approach to measuring APT burden on a local, national, and international level. This will help healthcare professionals, researchers, and policy developers understand the factors that contribute to overprescribing of APT medication (such as gender, ethnicity, nature of residence, level of ID and presence of co-occurring mental illness and/or other neurodevelopmental conditions), and develop targeted measures designed to mitigate such overprescribing. This study's findings will also provide a better understanding of the impact of the COVID-19 pandemic, and whether it did lead to a change in APT patterns. Where increased APT prescribing during the pandemic is observed in some healthcare trusts relative to others, factors contributing to such inter-trust differences can be explored.

Before similar methodology can be used to inform future studies, it is necessary to conduct this feasibility study to ensure that the study procedures allow the objectives to be met.

The feasibility objectives of the study are to evaluate whether it is:

(1) Feasible to identify PwID who have been prescribed ≥2 APTs over 7 years retrospectively.
(2) Possible to obtain a complete data set for each patient identified and therefore be able to explore the prescribing patterns across the eight sites.
(3) Feasible to quantify APT prescribing in PwID as Chlorpromazine equivalent dose values across different Healthcare Trusts in England and Wales.

The main objectives of the study are to investigate:

(4) Yearly and overall prescribing patterns among PwID [with or without mental health reasons (co-occurring mental health conditions)] in receipt of ≥2 forms of APT over time.
(5) How multiple APT prescribing has changed between 2017 and 2023 using chlorpromazine equivalent dose values, in PwID with mental health and no mental health indications.
(6) The impact of the COVID-19 pandemic (and corresponding lockdown restrictions in England and Wales) on multiple APT prescribing among PwID.
(7) Whether the SPC tool be utilised to track yearly APT prescribing among PwID receiving multiple forms of APT, and monitor variation between services (sites) and patient groups (e.g., psychiatric co-morbidities; challenging behaviour).

## Methods and analysis

### Study design

This is a multi-site cross-sectional study, involving data collection from specialist adult ID services based within eight NHS healthcare trusts across England and Wales over a seven-year time span (2017–2023). The eight trusts serve areas comprising of differing levels of urbanisation and pandemic-related lockdown restrictions to allow for the comparison of the impact of the COVID-19 pandemic on multiple APT prescribing among PwID.

The participating trusts are listed below, alongside their overall estimated catchment area:

• North East London NHS Foundation Trust (4.3 million) (16).
• Cheshire and Wirral Partnership NHS Foundation Trust (1 million) (17).
• Central and North West London NHS Foundation Trust (3 million) (18).
• Cornwall Partnership NHS Foundation Trust (450,000) (19).
• Coventry and Warwickshire Partnership NHS Trust (1 million) (20).
• Hertfordshire Partnership University NHS Foundation Trust (400,000) (21).
• Leicestershire Partnership NHS Trust (1.1 million) (22).
• Swansea Bay University Health Board (390,000) (23).

### Patient eligibility criteria and identification

#### Inclusion criteria

Patients must satisfy all the following criteria to be enrolled in the study:

(1) Having received a psychiatric review by specialist adult ID services within the last year^1^
(2) A diagnosis of ID
(3) Being under the care of specialist adult ID services within the last year of the date of interest
(4) In receipt of ≥2 APTs [oral and/or IM injectable (depots)]

^1^For a patient to be included on 1st June 2017, data is to be extracted from the most recent psychiatric review within the period between 1st January 2017–31st December 2017.

#### Exclusion criteria

Patients who meet any of the following criteria will be excluded from study participation:

(1) Under the age of 18 years
(2) Patients treated with Clozapine

Patients treated with Clozapine are excluded from the study as the drug is primarily used to treat specifically treatment-resistant Schizophrenia, whereby patients have been insufficiently responsive and/or intolerant of other APTs (24). Therefore, there would be a high likelihood that a patient treated with Clozapine has an appropriate diagnosis. Furthermore, those prescribed Clozapine are more likely to receive a more enhanced service due to the drug's side effect profile and the requirement for frequent blood monitoring (24), which is likely to differ from most PwID who are prescribed other antipsychotics, who encountered a lack of physical health reviews during the COVID-19 pandemic (9).

**Figure 1:**
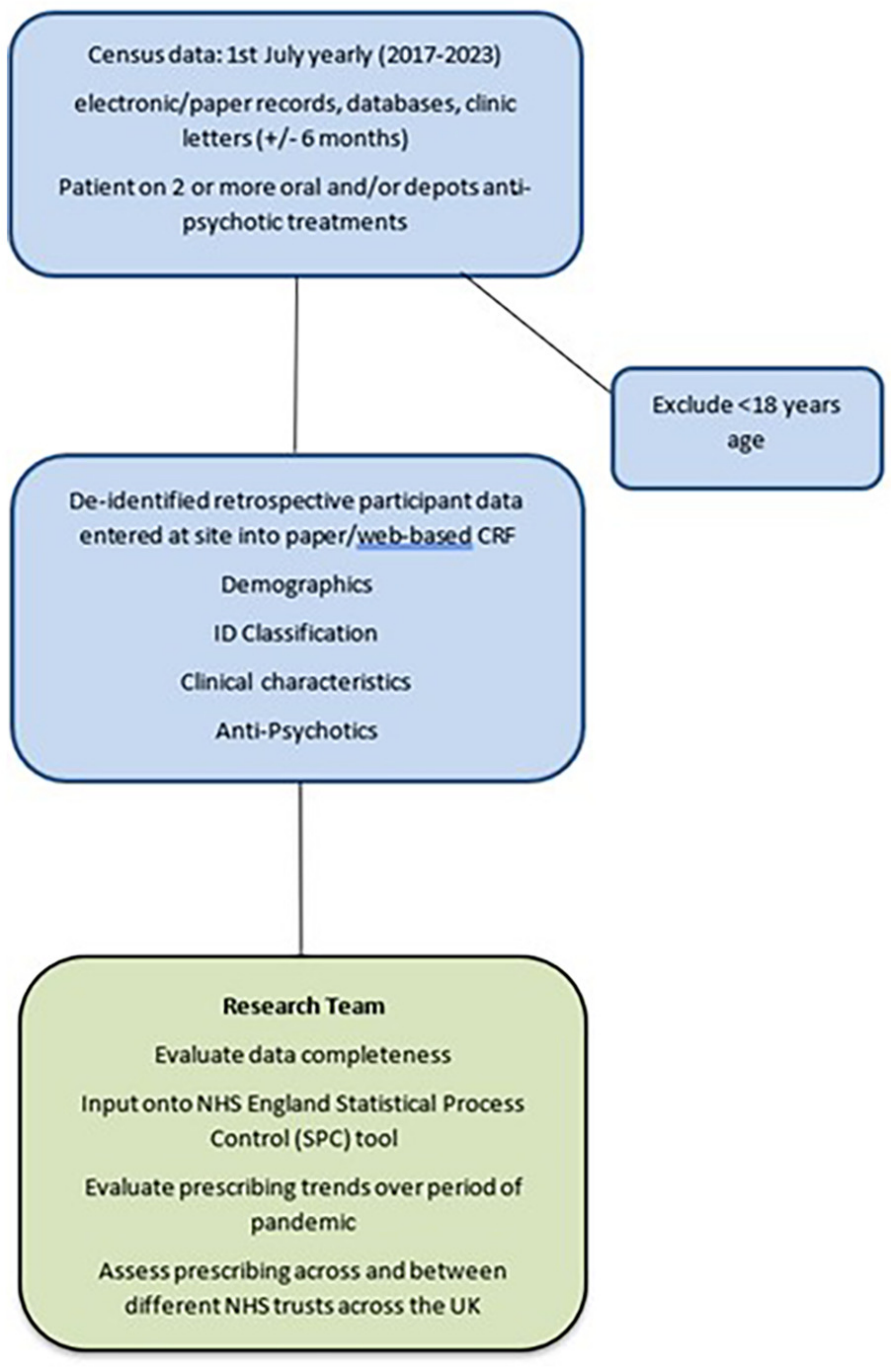
Study flow chart.

This is a retrospective multi-centre cohort study whereby patients are de-identified at site and thus patient consent is not required. At each site, the direct clinical care team (consultant, trainee doctor or specialist nurse) or a research nurse will identify 7–10 adult ID patients on their caseloads for each of the census dates. The patients who were prescribed ≥2 APTs will be identified from the paper-based clinic letters, inhouse databases (e.g., clinical portal bespoke)/electronic systems (e.g., SystmOne, Rio, Care Notes). Data will be extracted from the patient's most recent clinic letter to the census date (1st July of specified year), either up to 6 months before or 6 months, i.e., for July 2017, between 1st January 2017 and 31st December 2017. See Figure 1 for the study flow chart.

A study ID will be generated from REDCap, and only the routine care team (or research nurse) will have access to the code and patient identifier, to perform repeat entry for the same record, if required. As the data will be anonymised (de-identified) at site by the clinical team, patient consent is not required. If a patient is identified in consecutive years as meeting the inclusion criteria, they will be recorded as a separate record, with a unique ID, as this is not a longitudinal study so patients will not be tracked over time.

### Outcome measures

Data for patients will be collected using standardised data entry forms, for each of seven consecutive years (2017–2023). Data collected (Table 1) will include basic demographic details (age, gender, ethnicity, residential circumstances), clinical data (ID classification, genetic variants, medical co-morbidities, epilepsy, neurodevelopmental conditions, neurodegenerative conditions, psychiatric co-morbidities, challenging behaviour), and data on the APT (first/second generation, dose, units, frequency, route of administration, reason for prescribing).

**Table 1:** Data collection at each yearly time point (1st July 2017–2023).

			Timepoints yearly 01/07/2017–01/07/2023^a^
Caseload number on 2 or more antipsychotic treatments identified			
Date of nearest clinic letter			x
Demographics			
Age	Date of birth (month/year)		x
Gender	Male		x
	Female		x
	Gender non-conforming		
Ethnicity	Asian, Asian British, Asian Welsh		x
	Black, Black British, Black Welsh, Caribbean or African		x
	Mixed or multiple ethnic groups		x
	White		x
	Other ethnic group		x
Place of residence	Family home		x
	Residential home		x
	Supported living		x
	Living independently		x
	Other (describe)		x
ID classification			
ID classification	Mild		x
	Moderate/Severe/Profound		x
Genetic variants	Fragile X syndrome		x
	Down syndrome		x
	Tuberous sclerosis complex		x
	Developmental and/or epileptic encephalopathy		x
	Other		x
Clinical characteristics			
Co-occurring medical conditions^b^	Obesity		x
	Constipation (regular laxatives)		x
	Cerebral palsy and other paralytic syndromes		x
	Osteoporosis		x
	Epilepsy		x
	Metabolic syndromes	Diabetes	x
		Hypertension	x
		Dyslipidaemia	x
		Cardiovascular disease	x
	Other		x
Neurodevelopmental conditions	Autism spectrum disorder (ASD)		x
	Attention deficit hyperactivity disorder (ADHD)		x
Neurodegenerative conditions	Dementia		x
Co-occurring psychiatric conditions	Psychosis (Schizophrenia/schizoaffective disorder, bipolar affective disorder, severe depression with psychotic conditions or other		x
	Depressive disorders		x
	Anxiety disorders (obsessive compulsive disorder (OCD), Post-traumatic stress disorder (PTSD) or other		x
Challenging behaviour	Yes/no/not recorded		x
Antipsychotic drugs			
First generation APTs (most common brand name)^c^	Chlorpromazine (Largactil)—Levomepromazine (Nozinan)—Pericyazine (Neulactil)—Prochlorperazine (Stemetil)—Promazine—Trifluoperazine (Stelazine)—Benperidol (Frenactyl)—Haloperidol (Haldol)—Flupenthixol (Depixol; Fluanxol)—Zuclopenthixol (Clopixol)—Pimozide (ORAP)—Sulpiride		x
Second generation APTs (most common brand name)^c^	Amisulpride (Solian—oral route)—Aripiprazole (Abilify)—Asenapine—Cariprazine—Lurasidone—Olanzapine—Paliperidone—Quetiapine—Risperidone		x
Other^c^	Pipothiazine—Penfluoridol—Fluphenazine—Thioridazine—Other (free text)		x
Reason for each APT prescribed	Psychotic disorder		x
	To manage specific behavioural challenges/agitation		x
	To augment other psychotropic medication		x
	Anxiety disorder		x
	Other		x
	Not clearly indicated		x

^a^ Time-point: Most recent clinic letter to the 1st of July of the year under study; 6 months either side (e.g., 01/06/2017: between 1st January 2017 and 31st December 2017).

^b^ Co-occurring medical conditions, based on those most commonly reported in patients with ID.

^c^ Dose, units, frequency, and route of administration are recorded for each APT prescribed.

Data will be extracted from multiple sources for each patient by the clinical team. Source data will come from paper-based clinic letters, inhouse databases (e.g., clinical portal bespoke)/electronic systems (e.g., SystmOne, Rio, Care Notes). The yearly census date is the 1st of July and the patient's most recent information, and clinic letter will be identified (up to 6 months before or 6 months after the census date). Collected data will be anonymised at site by the clinical team and/or research nurses.

Data will be entered directly onto a study specific instance of REDCap (Research Electronic Data Capture), a secure web-based database. De-identified data will also be transferred on the SPC tool to evaluate and track the prescription changes over time. Only anonymised (de-identified) data will be available to the University of Plymouth research team/data processors for analysis. As the data is de-identified at site before being entered into the database, each year will be treated as independent even if patients are eligible year on year. There will be no patient linkage across time.

### Dose equivalent methods

To account for the different treatment doses of APT, data will be aggregated into a standardized form. Each patient's oral and long-acting IM injectable APT regime will be represented by their chlorpromazine equivalent dose values (14, 25), which offers a means of standardization for APT (14).

Chlorpromazine is a commonly used APT and a CPZ-equivalent dose is defined as a dose of antipsychotic that is comparable to 100 mg of CPZ. This is commonly used in clinical and research settings. It provides a universal language of APT burden that is readily understandable for healthcare professionals, researchers, policy makers, and the lay public alike.

There is no accepted standard for equivalence calculation and many methods have been documented with associated strengths and limitations. The study will utilise the Defined Daily Dose (DDD) equivalence method, highlighted in Leucht's 2016 paper, ‘Dose equivalents for antipsychotic drugs: The DDD method’ (25). This study acknowledges the limitations of all dose equivalence methods as highlighted by Patel et al. (26) including minimum effective dose, dose response curve, DDD and various consensus methods. The Leucht's DDD method, including equivalence tables, has been selected as a consistent approach to calculating equivalence. It always provides a method for comparing antipsychotic equivalence between first- and second-generation APTs. The authors highlight that this study is not intended to test equivalence methods, only to apply a consistent approach to calculation.

To facilitate conversion of APT to chlorpromazine equivalent dose values, a pre-programmed script in R/Stata will be utilised, following relevant conversion formulas [as per that included in the supplementary table that accompanies the Leucht et al. (25) article].

### Target sample size and justification

As this is a feasibility study no formal power calculation has been performed. The aim is for each site to collect between 7 and 10 patient's data per annum. Hence, the estimated study size will be approximately 392–600 patients for the seven-year period. It is estimated that there will be a high number of PwID receiving multiple oral and depot APTs each year which was estimated after discussion with the eight sites of their caseloads and the numbers expected on at least two oral and depots APTs and the duration they have been on review lists.

### Data analysis (statistical analysis plan)

The characteristics of the patients will be summarised descriptively, using means (SD) or number (%). Feasibility outcomes will be summarised by a descriptive analysis according to number and level of data completeness (expressed as a percentage value), including reporting the number of identified PwID who have been prescribed more than 2 APTs yearly over 7 years (Objective 1), the number and proportion (%) of PwID with complete data on oral and long-acting intra-muscular injectable APTs for each of the 7 years (Objective 2), and the number and proportion (%) of identified PwID at each site with calculable chlorpromazine equivalent dose values (14, 25) at each census date for each of the 7 years of interest (Objective 3).

To explore if APT prescribing has changed between 2017 and 2023 using chlorpromazine equivalent doses (Objective 4), the equivalent dose values per PwID in receipt of multiple APTs will be calculated and summarised using mean (SD) and median (IQR), where appropriate, by site and year. A mixed effects regression model will be used to determine changes in chlorpromazine equivalent dose values across time, including year as a fixed covariate and site as random effect. A subgroup analysis will explore these trends, by including as a fixed effect, the presence of mental health indications (Objective 5). To explore the impact of the COVID-19 pandemic on multiple APT prescribing among PwID (Objective 6), a time varying COVID variable will be added to the regression model.

The SPC tool will be used to compare trends in multiple APT prescribing both within and across participating healthcare trusts and explore if the SPC tool (10) can be utilised to track yearly oral and depots antipsychotic treatment prescribing and monitor variation between services and patient groups (Objective 7).

### Patient and public involvement

The study team includes Mrs Paula McGowan OBE who is an expert by experience. Her work in improving awareness to APT prescribing in PwID is well known globally following the sad loss of her son Oliver to an APT side effect (https://www.olivermcgowan.org/), resulting in mandatory training for all health and social care staff in ID and Autism. Paula is a co-applicant on the grant awarded and is a member of the monthly SMG for managing the study from beginning to end; she has also been involved in the development of the protocol.

### Ethics

The study was reviewed and approved by the University of Plymouth Faculty Research Ethics and Integrity Committee and Health Research Authority on 24/08/2023 and 07/09/2023 respectively (reference 23/HRA/3635).

The data arising from the study will be owned by the Sponsor. On completion of the study, the data will be analysed and tabulated, and a Final Report prepared. This report will be submitted to the Sponsor and Funder (Baily Thomas Charitable Fund) and will be accessed on request by contacting PenCTU. Participating investigators will not have rights to publish any of the study data without the permission of the CI and Sponsor.

## Results

The results section in this paper will outline the practical challenges that have been encountered when setting up the study.

### Trial status

At the time of submission, the current approved protocol version is V1.0 16.08.2023. The first site received green light authorisation on 30.11.2023 and patient identification commenced thereafter. The data collection end date is 01.04.2024.

### Administrative and technical

In the initial stages of the study, discussions were held surrounding whether patients prescribed Clozapine should be excluded from data collection. It was confirmed that these patients should be excluded, and the reasons for this are described in the methods and analysis section. As this was confirmed prior to the study's submission to the HRA, this was included in the protocol and therefore was made clear to sites in the protocol and during the site initiation visits. Once the study received HRA approval and sites started to open, it was queried whether patients who had been prescribed Promethazine Hydrochloride for agitation were eligible. It was noted that whilst the medication is not often prescribed for that indication, it is nevertheless an antipsychotic and when prescribed for agitation, patients could be included in the study. This was then fed back to the participating site.

As the study is a cross-sectional study that occurs over 7 annual time-points, the 1st July was identified as the census date for each yearly timepoint. This meant that sites have been asked to select the information and clinic letter that that occurred as close to this date as possible, though noting that data can be extracted from any point between 1st Jan–31st Dec for each timepoint. Originally, the 1st January was selected as the census date, but discussion within the trial management group found that this date may not have been the most efficient due to it being around Christmas and New Year. The census date of 1st July was selected as the mid-point of that year, so that data could be extracted from anywhere from 1st January–31st December, so long as the patient had had a psychiatric review in that year timepoint of interest.

Administrative and technical practical challenges such as those described above are bound to occur during the study's progress, and often participating sites may have queries that are perhaps not clarified into the study protocol. As the study is a feasibility study, practical challenges allow the study team to learn from them and take them into consideration when developing the protocol for a future definitive study.

### Identification

Another practical challenge that has occurred during the set-up of the study relates to the differing processes in discharging patients under each ID service. If an ID service is more likely to discharge a patient from their service sooner, it may result in having less eligible patients for the study. However, if the same patient remains under the ID service year on year, then there is the potential that the patient will be eligible to be recorded for each of the yearly timepoints and will therefore contribute to the site's identification target multiple times. This can then therefore lead to a difference in the number of eligible patients identified at each site. Though this is something that the study team do not have control over, it perhaps could be considered when confirming the recruitment targets for each study site.

### Research implementation

Implementing the outcome measures into the study protocol was another practical challenge that occurred during the study set-up. It is important that the outcome measures accurately reflect the study's objectives, and therefore a method had to be selected that allowed the APTs to be converted into equivalent doses so that the main objectives of the study could be tested. Chlorpromazine equivalent dose values were selected as they are commonly used in clinical and research settings, and therefore readily understandable for healthcare professionals, researchers and policy makers. Similarly, there is no accepted standard for equivalence calculation, and therefore Leucht's DDD (25) equivalence method was selected as it provides a method for comparing first- and second-generation APTs, which ensures that a consistent approach is applied to the calculation.

### Dissemination

The study will be reported in a manuscript that will be submitted to a peer-reviewed medical journal as open access. The group will also present results at relevant national and international conferences relevant to the mental health needs of adults with intellectual disability, such as the Royal College of Psychiatrist's Faculty of Intellectual Disability annual conference. Findings will also be presented to relevant ID organisations, including the Learning Disability National Professional Senate, The Challenging Behaviour Foundation, Speakup Self Advocacy, and Mencap. In addition to presenting the findings, reports will be provided in both standard and easy-read formats. Following the presentation, attendees will be asked to complete a brief semi-structured questionnaire to provide detailed feedback. This will help identify the views and priorities of the various stakeholder groups and help inform additional analyses to be incorporated into the final analysis and corresponding report.

## Discussion

There is a clear rationale for developing a database that establishes the antipsychotic prescribing patterns across specialist adult ID services, so that trends and patterns can be identified and further evaluated to improve the healthcare of PwID. Previous data has reported on prevalence prescribing rates, rather converting the APTs into dose equivalent values which can be input into the SPC tool to evaluate and measure ID specialist use of APTs in PwID across the UK. Furthermore, previous research has tended to report on entire clinical populations of PwID, rather than specifically a focus on those in receipt ≥2 different forms of APTs. This, along with the fact that there is currently no clinical rationale or evidence to support ≥2 different APTs being prescribed, supports the idea that there a need for a database to address this issue. However, the development of a de-identified database can present a series of unique benefits and challenges.

The limitations of the study are inherent to the use of routine health data. The use of routinely collected data means that the data quality and data completeness between sites might be vastly different. Site teams are encouraged to extract data from multiple sources, including clinic letters, inhouse databases and electronic or paper note systems. However, some of the data being collected might not be evident in any of these places and could potentially be difficult to find. This was acknowledged as a potential difficulty during study development and one of the feasibility objectives of the study is to evaluate whether it is indeed feasible to obtain a complete (or near complete) data set for each patient. An additional limitation of the study is that some patients may be in receipt of one or more of their APTs on a short-term basis.

The use of routinely collected data can result in a reduced burden for staff at participating NHS sites. Staff are not required to conduct any questionnaires with participants for this study and are advised to only use what is already available. Due to the fact the data is routinely collected and is de-identified at site level before being entered into the database, site staff are not required to receive consent from patients before their data is included, which again reduces the workload associated with the study which can be beneficial in sites where capacity is limited. Similarly, the non-requirement for consent procedures can also have great advantages in research with PwID who may be difficult to enrol in studies that require consent. It can also ensure that certain hard-to-reach patient groups are not missed, such as those who are unable to consent for themselves and do not have anyone who is able to provide advice on their behalf, which can lead to a lack of representation of this patient group in the study results.

This feasibility study is intended to inform decisions about the design of a wider registry that would involve antipsychotic prescribing data for patients across multiple sites nationwide. Once the prescribing patterns of those prescribed ≥2 APTs are further understood, it may allow for the opportunity to progress to obtain further understanding of the patterns across the country for those on one APT. By using the SPC tool as a validated means of plotting data, it can be utilised to see if it is possible to track yearly APT prescribing among PwID receiving multiple forms of APTs and monitor variation between services and patient groups (e.g., co-occurring mental health conditions, challenging behaviour) or not. This could allow researchers to identify trends and patterns of variation. If the use of the SPC tool proves successful, then the data collected across multiple participating trusts across the UK could be inputted into the SPC tool and used to evaluate and measure ID specialist service use of APTs in PwID nationwide.

## Data Availability

The original contributions presented in the study are included in the article, further inquiries can be directed to the corresponding author.
